# Supplementary material for: Carbon-wise utilization of lignin-related compounds by synergistically employing anaerobic and aerobic bacteria
Source: Biotechnol Biofuels Bioprod. 2024 Jun 8;17:78. doi: 10.1186/s13068-024-02526-0 (PMC11161944; doi:10.1186/s13068-024-02526-0)
Supplement: Supplementary file 1 — Supplementary Material 1. Cultivating A. woodii on vanillate as a sole organic carbon source. Cultivating A. woodii on ferulate and coumarate as a sole organic carbon source. Cultivating A. woodii on guaiacol. Sequence of the knock-out cassette. Table S1: Stoichiometric equations of vanillate, syringate, and ferulate utilization by A. woodii according to Bache and Pfennig. Table S2: The production of PCA and acetate by A. woodii when cultivated on vanillate as a sole organic carbon source. Standard deviations describe the differences between the duplicates. Table S3: The production of caffeate, dihydrocaffeate, and acetate by A. woodii when cultivated on ferulate as a sole organic carbon source. Standard deviations describe the differences between the duplicates. Table S4: The production of phloretate and acetate by A. woodii when cultivated on coumarate as a sole organic carbon source. Standard deviations describe the differences between the duplicates. Table S5: The production of catechol and acetate by A. woodii when cultivated on guaiacol as a sole organic carbon source. The standard deviations describe the differences between the duplicates. Table S6: List of the used primers and their names. Figure S1: A correlation curve between OD600 and CDW in ADP1. Figure S2: A A. woodii cultivated in acetobacterium medium with A ferulate or B coumarate as a sole organic carbon source. The fructose present at the beginning of the cultivation is a carry-over substrate coming from the inoculation. Error bars in both graphs are describing the differences between the duplicates. The error bars indicate the standard deviations from the average values of the biologically independent duplicates. In some cases, the error bars are smaller than the size of the marker. Figure S3: The growth of ADP1 on ferulate or caffeate and dihydrocaffeate modeling the aromatic metabolites obtained when A. woodii is cultivated on ferulate. Used concentrations are A 5 mM, B 7.5 mM, and C 10 mM. The [file 13068_2024_2526_MOESM1_ESM.docx]

Supplementary materials

**Carbon-wise utilization of lignin-related compounds by synergistically employing anaerobic and aerobic bacteria**

Ella Meriläinen, Elena Efimova, Ville Santala, Suvi Santala*

Faculty of Engineering and Natural Sciences, Tampere University, Hervanta Campus, Korkeakoulunkatu 8, 33720 Tampere, Finland

*corresponding author: [suvi.santala@tuni.fi](mailto:suvi.santala@tuni.fi)

Supplementary notes

**The correlation between OD600 and cell dry weight (CDW) in ADP1**

To determine the correlation between the optical densities measured at 600 nm (hereafter OD600s) and cell dry weights (CDW) in ADP1 cultivations, we cultivated ADP1 wild type (WT) on 1 X MSM with either 20 mM p-hydroxybenzoate or 100 mM acetate as a carbon source. The cultivation volume was 50 mL, and the starting OD600 was set to be 0.1. The cultivations were kept in +30 °C/300 rpm until the OD600 was over 2.0. After the OD600 measurements, the cells were harvested and CDWs were determined by freeze drying the cells for 24 h. The correlation curve between OD600s and CDWs is presented in Supplementary Fig. S1.

**Cultivating *A. woodii* on vanillate as a sole organic carbon source**

The cultivation of *A. woodii* in acetobacterium medium on 6, 12 and 18 mM vanillate as a sole carbon source was done to determine if the experimental molar yields have notable difference with the literature values (Table S2). All vanillate was demethylated in every cultivation, and protocatechuate (PCA) and acetate were produced. The obtained maximum OD600s were positively correlated with the initial vanillate concentrations, and no growth inhibition was observed with any concentrations. When *A. woodii* was cultivated without any added carbon sources, carry-over substrates from the cell inoculation resulted in minor cell growth (OD600 < 0.1).

**Cultivating *A. woodii* on ferulate and coumarate as a sole organic carbon source**

To study what metabolites and yields could be obtain by cultivating *A. woodii* on ferulate or coumarate and how the obtained results compare to the existing literature, *A. woodii* was cultivated on 13 mM ferulate or in 14 mM coumarate in acetobacterium medium in N_2_-CO_2_-headspace. Acetate, caffeate (3-(3,4-dihydroxyphenyl)-2-propenate) and dihydrocaffeate were obtained as metabolites, when *A. woodii* was cultivated on ferulate (Fig. S2A). The molar yields of dihydrocaffeate and acetate from ferulate were 0.70 ± 0.24 mol/mol and 0.66 ± 0.14 mol/mol, respectively (Supplementary Table S3). The conversion of caffeate to dihydrocaffeate was observed to occur at notably different rates. When *A. woodii* was cultivated on 14 mM coumarate in N_2_-CO_2_-headspace, phloretate (3-(4-hydroxyphenyl)propionate) and acetate were produced (Fig. S2B) with molar yields of 0.20 ± 0.05 mol/mol and 0.19 ± 0.04 mol/mol, respectively (Supplementary Table S4).

Unlike in the literature, a complete conversion of ferulate into dihydrocaffeate with a molar yield of 1.0 mol/mol (1) was not achieved, although all ferulate was consumed. Dihydrocaffeate can be obtained from ferulate if ferulate’s methoxyl group is demethylated into hydroxyl group and the double bond in the acrylate side chain is reduced. The conversion of ferulate into caffeate requires less modifications: the caffeate molecule still contains the double bond in the side chain, and therefore only the methoxyl group of the ferulate must be demethylated. It can be hypothesized that the lower molar yield of dihydrocaffeate obtained in this study was resulted from the incomplete conversion of caffeate into dihydrocaffeate, most likely caused by the additional electron acceptor CO_2_ (2). The obtained molar yield of acetate from ferulate was higher than the literature value 0.5 mol/mol, indicating that some of the available electrons from the ferulate’s methoxyl groups or carry-over fructose were donated to CO_2_ fixation.

Only 2.6 mM coumarate was consumed during the 7-day cultivation period. However, theoretically, no coumarate should have been consumed, due to the lack of electron donor groups in coumarate molecule. The reduction of coumarate into phloretate and the appearance of acetate can be explained by the carry-over substrate fructose providing electrons for the double bond reduction and CO_2_ fixation.

**Cultivating *A. woodii* on guaiacol**

To determine the possible differences between the existing literature values and experimental data, *A. woodii* was cultivated on 6, 11 or 18 mM guaiacol in acetobacterium medium (Table S5). *A. woodii* was able to grow on guaiacol as a sole carbon source and no residual guaiacol was detected in the cultivations in the end. Catechol and acetate were detected as only end-products. When *A.* woodii was cultivated without any carbon source, the carry-over substrate fructose from the inoculation resulted only small production of acetate, 0.6 ± 0.1 mM and minor increase in biomass (final OD600 < 0.1 ± 0.0, increase from the initial OD600 only 0.05 ± 0.0).

The obtained OD600 values and the initial guaiacol concentrations were positively correlated, except in cultivations containing 18.1 mM guaiacol, where the biomass obtainment was similar to cultivations containing 11.4 mM guaiacol. Thus, the highest guaiacol concentration 18.1 mM seemed to hinder the biomass accumulation. This effect was also reported in the previous literature (3,4). However, the increase in guaiacol concentrations did not result in lower molar yields of catechol like reported previously (3). The molar yield of catechol obtained with 18.1 mM guaiacol was also noticeably higher (0.85 ± 0.0 mol/mol) than the catechol yield reported in literature when *A. woodii* was cultivated on 15 mM guaiacol (0.52 mol/mol) (3). The variances in the obtained molar yields of acetate was greater than in the molar yields of catechol, but the acetate concentrations are in the same range than reported in the literature (4).

**Cultivating ADP1 WT on acetate and catechol mixture**

To study how ADP1 WT consumes catechol and acetate when both substrates are present in the medium, ADP1 WT was cultivated on 6 mM catechol and 28 mM acetate in 1 x MSM medium (Figure S7). The cultivation volume was 30 mL, and the starting OD600 was adjusted to 0.3. The cultivation was done with RTS-8 Plus multi-channel bioreactor (Biosan, Latvia), in +30°C, using 2600 rpm, and the reverse spin interval was set to 3 seconds. The device measured OD600s in every 10 minutes. Catechol consumption begun faster, but eventually the bacterium was able to consume both substrates simultaneously (between 4-8 h).

**Sequence of the knockout cassette:**

acagtaaggagctgacgtaaccaattctcaaggtttgactgaccgttgcaatccgttttgccattgaggacgcttgtgcagtatgagtcaaaatctgcacagcatgctgataaaaaaacatgcctgcttcagtcactttagccggtctgaagccgcgttcaaatagctgaattcccaattcttcttcgagtttttgaatttgtcggctgaggggcggctgggcaatacacaacttttcagcagctttggaaatgctttgctcttcaaccacggtcacaaaatatctgaggtgtcttagttccatttatacgccctaattggttttatatacctttttagtatgcaaaaataccaaattgtttatcttttttattattacattaatttaaggtatgtaaatagtatttattgaaaagaagatggaccgatgtataaatcagtggaaactattttaatttatttgctttattaaagaggagaaattaattaatggcacagctatatttctactattccgcaatgaatgcgggtaagtctacagcattgttgcaatcttcatacaattaccaggaacgcggcatgcgcactgtcgtatatacggcagaaattgatgatcgctttggtgccgggaaagtcagttcgcgtataggtttgtcatcgcctgcaaaattatttaaccaaaattcatcattatttgatgagattcgtgcggaacatgaacagcaggcaattcattgcgtactggttgatgaatgccagtttttaaccagacaacaagtatatgaattatcggaggttgtcgatcaactcgatatacccgtactttgttatggtttacgtaccgattttcgaggtgaattatttattggcagccaatacttactggcatggtccgacaaactggttgaattaaaaaccatctgtttttgtggccgtaaagcaagcatggtgctgcgtcttgatcaagcaggcagaccttataacgaaggtgagcaggtggtaattggtggtaatgaacgatacgtttctgtatgccgtaaacactataaagaggcgttacaagtcgactcattaacggctattcaggaaaggcatcgccacgattaacggccgccaccgcggtggagctcggtacccggggatcctctagagcggacccgggaaagccacgttgtgtctcaaaatctctgatgttacattgcacaagataaaaatatatcatcatgaacaataaaactgtctgcttacataaacagtaatacaaggggtgttatgagccatattcaacgggaaacgtcttgctcgaggccgcgattaaattccaacatggatgctgatttatatgggtataaatgggctcgcgataatgtcgggcaatcaggtgcgacaatctatcgattgtatgggaagcccgatgcgccagagttgtttctgaaacatggcaaaggtagcgttgccaatgatgttacagatgagatggtcagactaaactggctgacggaatttatgcctcttccgaccatcaagcattttatccgtactcctgatgatgcatggttactcaccactgcgatccccgggaaaacagcattccaggtattagaagaatatcctgattcaggtgaaaatattgttgatgcgctggcagtgttcctgcgccggttgcattcgattcctgtttgtaattgtccttttaacagcgatcgcgtatttcgtctcgctcaggcgcaatcacgaatgaataacggtttggttgatgcgagtgattttgatgacgagcgtaatggctggcctgttgaacaagtctggaaagaaatgcataagcttttgccattctcaccggattcagtcgtcactcatggtgatttctcacttgataaccttatttttgacgaggggaaattaataggttgtattgatgttggacgagtcggaatcgcagaccgataccaggatcttgccatcctatggaactgcctcggtgagttttctccttcactacagaaacggctttttcaaaaatatggtattgataatcctgatatgaataaattgcagtttcatttgatgctcgatgagtttttctaagcatgcggagctggtatgtaaatagagatgacacttcatcagtgttcatctcttacgtttatggtatttctttattttccgttttcatccaaaggttgatgacatgatagataaaagtgcagcgaccctaacggaagcgctctcccagatccacgacggtgccaccatcctgattggtggttttggaacagccggccaacccgccgagctgattgacggactgattgaactaggtcgcaagaacctgaccatcgtcagcaacaacgccggcaatggagactatgg

Supplementary tables

*Table S1: Stoichiometric equations of vanillate, syringate and ferulate utilization by A. woodii according to Bache and Pfennig* (1)

| Aromatic compound | Stoichiometric  equation | | | Molar ratio of acetate/substrate | Molar ratio of acetate/CH_3_-groups | |
| --- | --- | --- | --- | --- | --- | --- |
| Vanillate | | $4 vanillate+2 CO_{2}+2 H_{2}O \to4 PCA+3 CH_{3}COOH$ | 0.75 | | | 0.75 |
| Syringate | | $2 syringate+ 2 CO_{2}+2 H_{2}O \to2 gallate+ 3 CH_{3}COOH$ | 1.5 | | | 0.75 |
| Ferulate | | $2 ferulate+2 H_{2}O \to2 dihydrocaffeate+CH_{3}COOH$ | 0.5 | | | 0.5 |

*Table S2: The production of PCA and acetate by A. woodii when cultivated on vanillate as a sole organic carbon source. Standard deviations describe the differences between the duplicates.*

| Initial vanillate concentration (mM) | OD600* | Produced  PCA (mM) | Produced  acetate (mM) | Molar yield of PCA from vanillate | Molar yield of acetate from vanillate |
| --- | --- | --- | --- | --- | --- |
| 5.9 ± 0.3 | 0.2 ± 0.0 | 4.7 ± 0.8 | 5.6 ± 0.5 | 0.80 ± 0.18 | 0.95 ± 0.13 |
| 12.2 ± 0.7 | 0.3 ± 0.0 | 11.7 ± 0.9 | 8.3 ± 1.4 | 0.96 ± 0.17 | 0.68 ± 0.15 |
| 18.4 ± 0.3 | 0.4 ± 0.0 | 14.5 ± 0.7 | 9.7 ± 0.9 | 0.79 ± 0.05 | 0.53 ± 0.06 |

*The presented OD600 value is the highest measured value

*Table S3: The production of caffeate, dihydrocaffeate and acetate by A. woodii when cultivated on ferulate as a sole organic carbon source*. Standard deviations describe the differences between the duplicates.*

| Initial ferulate concentration (mM) | Maximum OD600 | Produced  caffeate (mM) | Produced dihydro-caffeate (mM) | Produced  acetate (mM) | Molar yield of dihydrocaffeate from ferulate | Molar yield of acetate from ferulate |
| --- | --- | --- | --- | --- | --- | --- |
| 12.8 ± 0.4 | 0.7 ± 0.1 | 5.6 ± 3.3 | 9.0 ± 2.9 | 8.4 ± 1.5 | 0.70 ± 0.24 | 0.66 ± 0.14 |

*Ferulate was a sole organic carbon source, but the inoculation of the cells also caused small amounts of carry-over fructose to be present in the medium.

*Table S4: The production of phloretate and acetate by A. woodii when cultivated on coumarate as a sole organic carbon source*. Standard deviations describe the differences between the duplicates.*

| Initial coumarate  concentration (mM) | Maximum OD600 | Produced  phloretate (mM) | Produced  acetate** (mM) | Molar yield of phloretate from coumarate | Molar yield of acetate from coumarate |
| --- | --- | --- | --- | --- | --- |
| 14.1 | 0.4 ± 0.0 | 2.9 ± 0.7 | 2.7 ± 0.5 | 0.20 ± 0.05 | 0.19 ± 0.04 |

*Coumarate was a sole organic carbon source, but the inoculation of the cells also caused small amounts of carry-over fructose to be present in the medium.

**Because the acetate concentration decreased slightly towards the end of the cultivation, the concentration of the produced acetate was determined by taking an average of the last three concentrations and subtracting the concentration obtained from carry-over acetate coming from the inoculation.

*Table S5: The production of catechol and acetate by A. woodii when cultivated on guaiacol as a sole organic carbon source. The standard deviations describe the differences between the duplicates.*

| Initial guaiacol concentration (mM) | OD600* | Produced catechol (mM) | Produced acetate (mM) | Molar yield of catechol | Molar yield of acetate |
| --- | --- | --- | --- | --- | --- |
| 5.9 ± 0.7 | 0.2 ± 0.0 | 4.9 ± 0.4 | 5.1 ± 2.2 | 0.83 ± 0.2 | 0.86 ± 0.5 |
| 11.4 ± 0.3 | 0.3 ± 0.1 | 9.7 ± 0.2 | 8.0 ± 1.1 | 0.85 ± 0.0 | 0.70 ± 0.1 |
| 18.1 ± 0.7 | 0.3 ± 0.0 | 15.3 ± 0.0 | 10.7 ± 0.4 | 0.85 ± 0.0 | 0.59 ± 0.0 |

*The presented OD600 value is the highest measured value

*Table S6: List of the used primers and their names*

| Name | Primer |
| --- | --- |
| CatB_P3 | ACAGTAAGGAGCTGACGTAACC |
| CatB_P4 | TTTTTATGATTTGAATTGGAGGCTGGGCGGTCCATCTTCTTTTCAA |
| CatC_P5 | CGATGAGTTTTTCTAAGCATGCGGAGCTGGTATGTAAATAGAGATGACACTTC |
| CatC_P6 | CCATAGTCTCCATTGCCGGC |
| Tdk_kanF | CCCAGCCTCCAATTCAAATCATAAAAAATTTATTTG |
| Tdk_kanR | CCAGCTCCGCATGCTTAGAAAAAC |

Supplementary figures


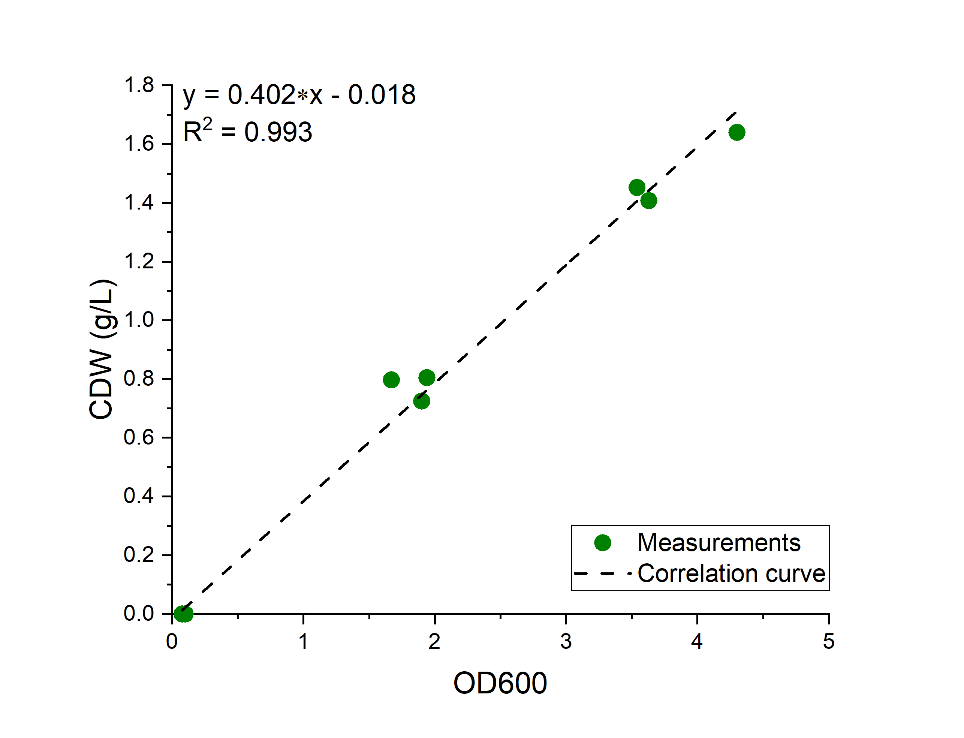


*Figure S1: A correlation curve between OD600 and CDW in ADP1.*


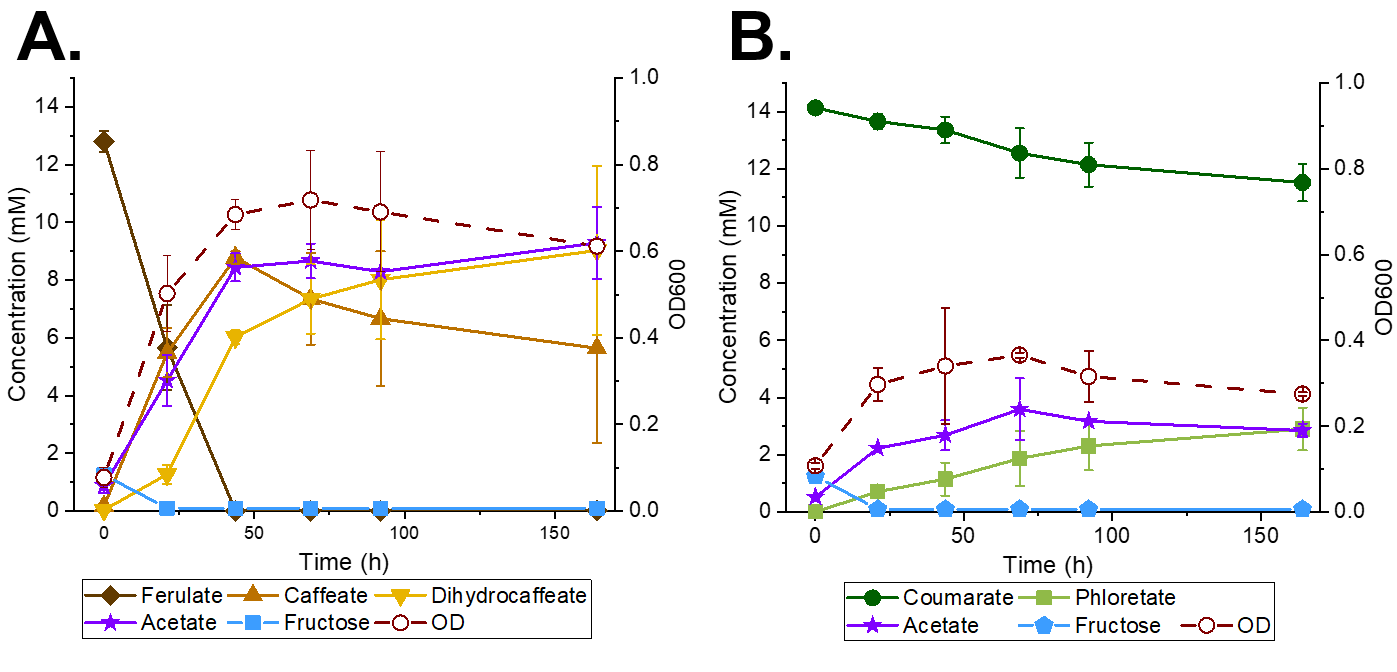


*Figure S2: A.) A. woodii cultivated in acetobacterium medium with A.) ferulate or B.) coumarate as a sole organic carbon source. The fructose present at the beginning of the cultivation is a carry-over substrate coming from the inoculation. Error bars in both graphs are describing the differences between the duplicates. The error bars indicate the standard deviations from the average values of the biologically independent duplicates. In some cases, the error bars are smaller than the size of the marker.*


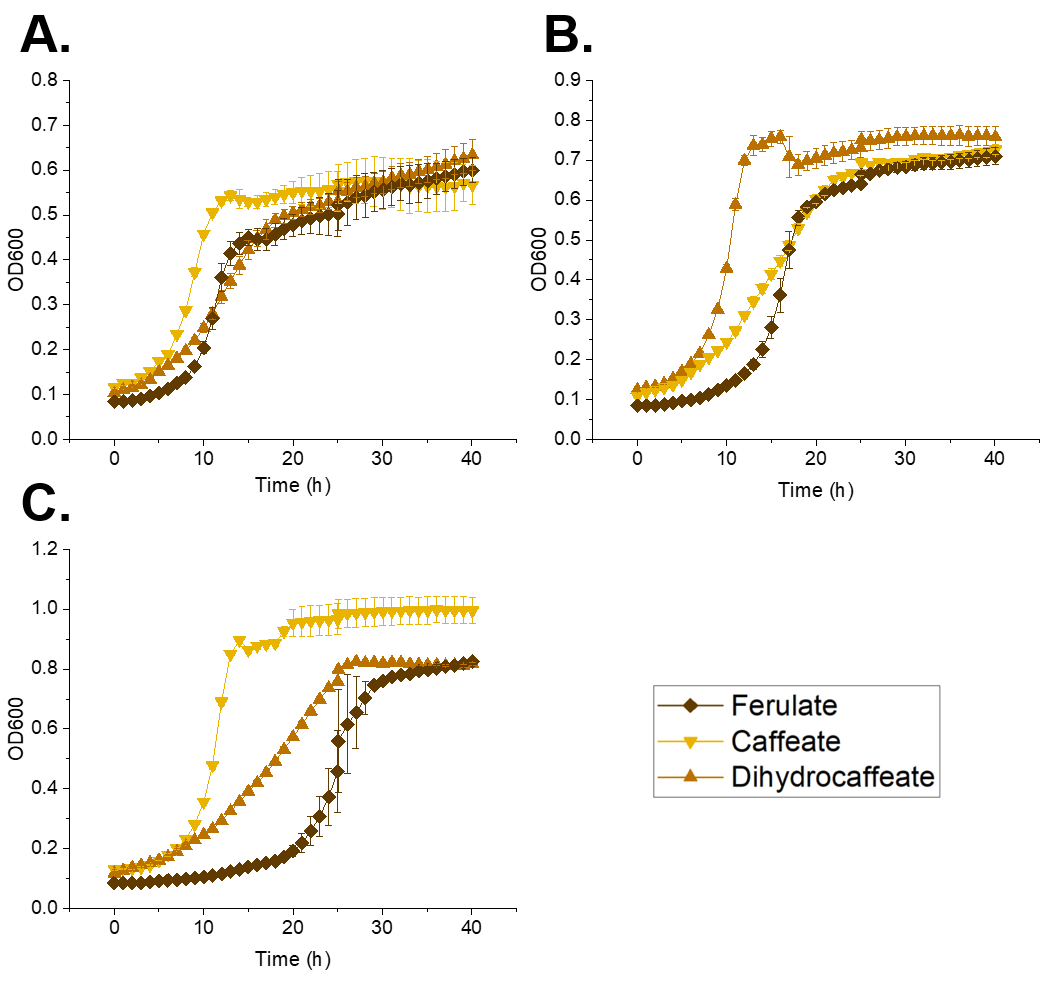


*Figure S3*: *The growth of ADP1 on ferulate or caffeate and dihydrocaffeate modelling the aromatic metabolites obtained when A. woodii is cultivated on ferulate. Used concentrations are A.) 5 mM, B.) 7.5 mM and C.) 10 mM. The error bars indicate the standard deviations from the average values of the biologically independent triplicates. In some cases, the error bars are smaller than the size of the marker.*

*
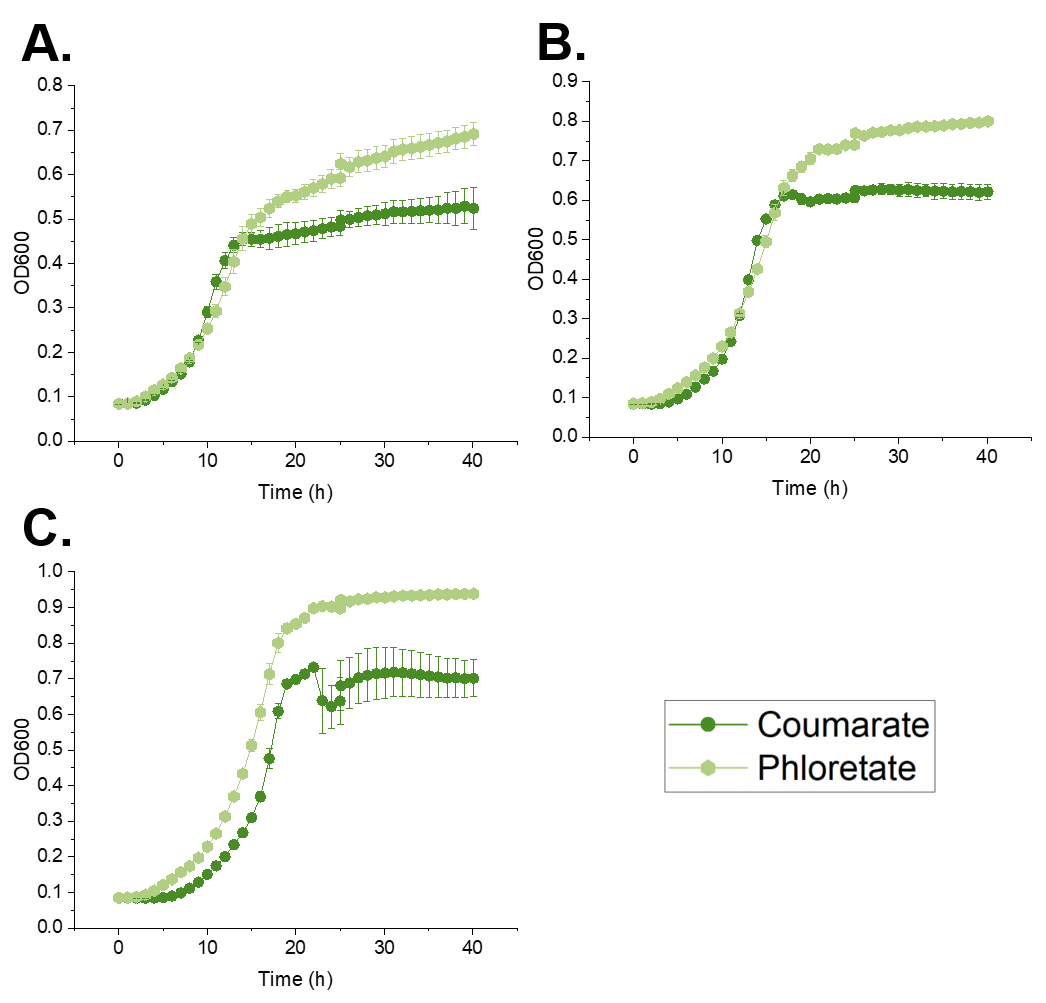
*

*Figure S4: The growth of ADP1 on coumarate or phloretate modelling the aromatic metabolites obtained when A. woodii is cultivated on coumarate. Used concentrations are A.) 5 mM, B.) 7.5 mM and C.) 10 mM. The error bars indicate the standard deviations from the average values of the biologically independent triplicates. In some cases, the error bars are smaller than the size of the marker.*

*Figure S5: The growth of ADP1 in MSM with catechol as a sole carbon source. The linear increase of OD600 in 10 mM cultivations was caused by the color change of catechol in the medium and therefore it is not representing the accumulation of biomass.* *The error bars indicate the standard deviations from the average values of the biologically independent triplicates. In some cases, the error bars are smaller than the size of the marker.*

*Figure S6: Experimental data of the three-phase one-pot coculture of A. woodii and ADP1ΔcatBC. In this graph, only the utilization and production of sugars and organic acids are presented. Glucose is supplemented in the medium at the beginning of the cultivation. Fructose and acetate peaks at the first dashed lined are caused by the inoculation of A. woodii. Please notice that compared to Fig. 5, the presented acetate concentration here describes the overall concentration of acetate, not only the acetate produced by A. woodii. In these growth conditions, ADP1 consumed acetate faster than glucose during the aerobic phase, which is potentially related to the complex medium composition and a long oxygen-depletion period; previously, ADP1 has been reported to consume acetate and glucose simultaneously* (5)*. DO = deoxygenation phase.*


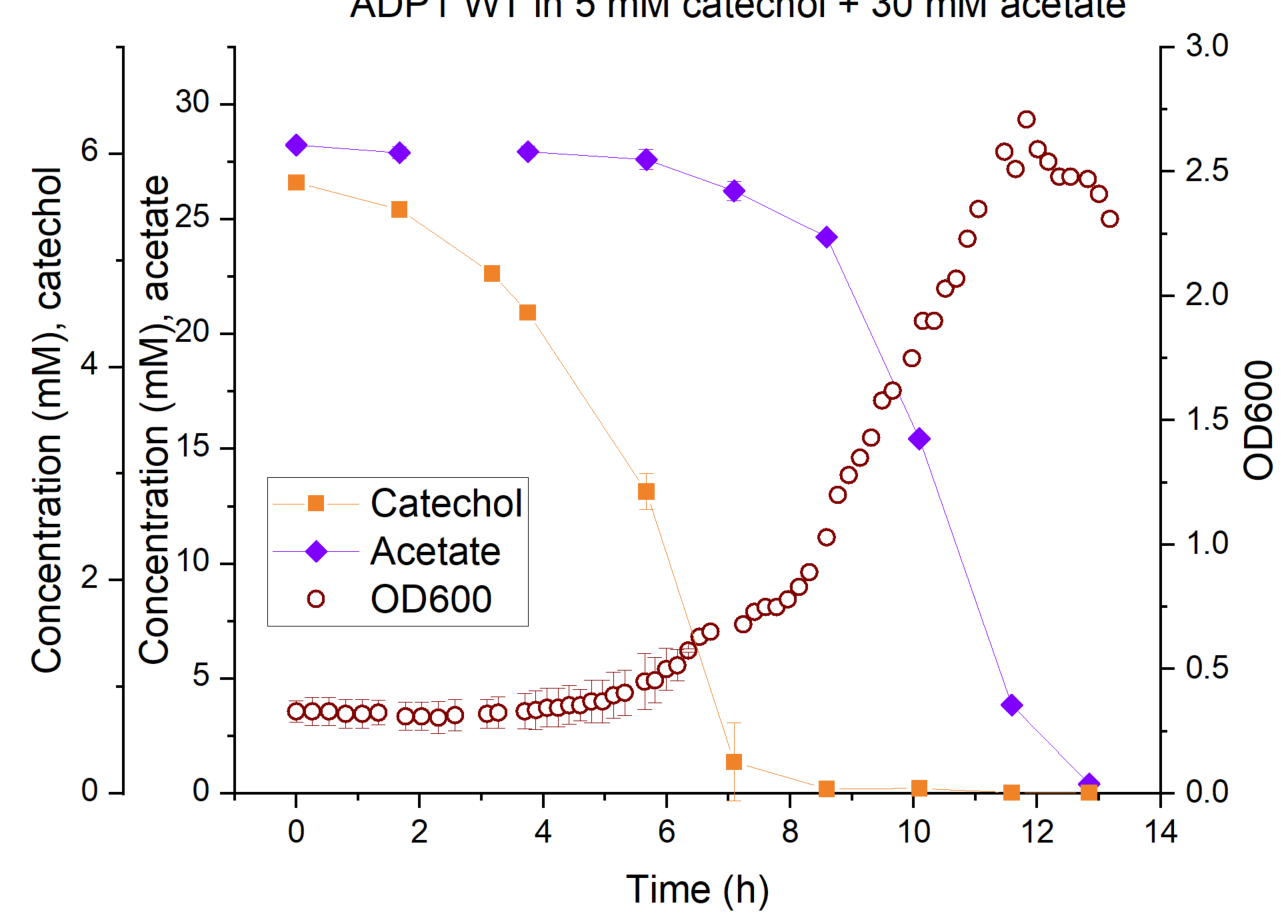


*Figure S7: Consumption of catechol and acetate by ADP1 WT. The error bars indicate the standard deviations from the average values of the biologically independent duplicates. In some cases, the error bars are smaller than the size of the marker. One of the duplicate cultivations was lost after 7 h, and thus the rest of the data points are describing values obtained from single cultivation.*

## References

1. Bache R, Pfennig N. Selective isolation of Acetobacterium woodii on methoxylated aromatic acids and determination of growth yields. Arch Microbiol. 1981;130(3):255–61.

2. Davies ET, Stephens GM. Effect of growth substrate and electron donor on hydrogenation of carbon-carbon double bonds by Acetobacterium woodii. Enzyme and Microbial Technology. 1998 Jul 1;23(1):129–32.

3. Kalil MS, Stephens GM. Catechol production by O-demethylation of 2-methoxyphenol using the obligate anaerobe, Acetobacterium woodii. Biotechnol Lett. 1997 Dec 1;19(12):1165–8.

4. Kalil MS, Zainatul`Asyiqin, Zaki M. Catechol Synthesis via Demethylation of Guaiacol by Anaerobic Bacterium Acetobacterium woodii DSM 1030. Pak J Biol Sci. 2002;5(11):1186–8.

5. Salcedo-Vite K, Sigala JC, Segura D, Gosset G, Martinez A. Acinetobacter baylyi ADP1 growth performance and lipid accumulation on different carbon sources. Appl Microbiol Biotechnol. 2019 Aug 1;103(15):6217–29.
